# Metabolic regulation of NK cell function: implications for immunotherapy

**DOI:** 10.1097/IN9.0000000000000020

**Published:** 2023-01-23

**Authors:** Hyogon Sohn, Megan A. Cooper

**Affiliations:** 1 Division of Rheumatology/Immunology, Department of Pediatrics, Washington University, St. Louis, MO, USA

**Keywords:** NK cells, immune memory, immunometabolism, immunotherapy, interferon-gamma, interleukin-15, cytokines

## Abstract

Natural killer (NK) cells are innate immune lymphocytes capable of rapidly responding to tumors and infection without prior sensitization. There is increasing interest and success in harnessing NK cell function for the treatment of disease, in particular cancers. NK cell activation is dependent on integration of signals through cytokine and germline-encoded activating and inhibitory receptors. The availability of metabolic fuels and pathways is required for NK effector functions including proliferation, killing, and production of interferon gamma (IFN-γ). An understanding of NK cell immunometabolism is thus essential for developing immunotherapy approaches that will allow for optimal effector functions in patients. Studies in mice and humans have demonstrated stimulation-dependent metabolic changes that are required for NK cell function. Here we review the most recent findings in NK cell immunometabolism relevant to disease models and translation to therapy of patients.

## 1. Introduction

Natural killer (NK) cells are innate lymphocytes that contribute to the immune response against malignancies and viral infections. NK cells act as a first line of defense due to the fact that they require no receptor gene rearrangement like B and T cells. NK cell immunotherapy for cancer is a rapidly evolving field, with clinical trials using allogeneic or autologous activated NK cells or chimeric antigen receptor (CAR) NK cells, as well as administration of cytokines and/or antibodies targeting NK cell effector activities ^[1–12]^.

Access to metabolic pathways and fuels is essential for immune cell function, making consideration of immunometabolism is important to optimize immunotherapy approaches. Tumor cells create a unique environment (tumor microenvironment [TME]) that has the potential to limit immune cell activity, including NK cells, in a variety of ways. For example, many tumor cells secrete anti-inflammatory cytokines or create a low glucose environment for infiltrating immune cells by frequently employing glycolysis to support their aberrant proliferation.

Metabolic requirements for many NK cell function have now been elucidated, as summarized in recent reviews in the field ^[13–16]^. While approaches to optimize NK cell metabolism in the context of immunotherapy are still uncertain, knowledge gained from basic studies in the mouse and human systems can be used to begin to design new approaches that consider NK cell metabolic requirements for activation. Here we discuss recent findings on metabolic requirements for NK cell function, and provide background and speculation on approaches to optimize NK cell performance in metabolically challenging environments.

## 2. Metabolic requirements differ by NK cell activation state and route of stimulation

Quiescent NK cells have few metabolic requirements, effectively utilizing glucose-driven oxidative phosphorylation (OXPHOS), similar to resting T cells ^[17,18]^. Activation leads to metabolic changes, influencing NK cells effector functions.

### 2.1 NK cell biology: activation and effector functions

NK cells can be activated by two main pathways: receptors and cytokines ^[19]^. Germline-encoded activating NK receptors (NKRs) in mice and humans recognize pathogen-associated ligands alone or in the context of MHC-I, and stress-induced self-ligands. Activating NKRs (ActR) signal by partnering with adapters (DAP12, DAP10, FcεRIγ, and/or CD3ζ) bearing immunoreceptor tyrosine-based activation motifs (ITAMs) ^[20–22]^. NK cells recognize the Fc fragment of antibodies through the activating CD16 (FcγRIII) receptor, signaling through CD3ζ and FcεRIγ ^[23–25]^. Inhibitory NKRs recognize self-MHC and act as a “brake” of NK response to healthy tissues through immunoreceptor tyrosine-based inhibitory motifs (ITIMs) in their cytoplasmic tail ^[26]^. Inhibitory NKRs are also critical for NK cell “licensing”, a process by which only NK cells capable of recognizing self MHC-I acquire functional competence ^[27]^.

NK cells constitutively express receptors for many cytokines, including interleukin (IL)-2, IL-12, IL-15, IL-18, and IL-21, allowing them to quickly respond to inflammatory signals ^[19]^. Cytokine signals activate transcription factors including STATs and NFκB, leading to a generally proinflammatory transcriptional program. These cytokine signals are important for NK cell interferon gamma (IFN-γ) production, a signal important for promoting MHC-I expression, differentiating T cells, and immune cells recruitment to the TME ^[28]^.

The ability of NK cell to kill targets, including tumor cells, is the result of a combination of receptor and cytokine signals. Cytokines are important for NK cell expansion and “arming” with cytotoxic machinery, including granzymes and perforin ^[29]^. On encountering a target cell, activation of NK cells is regulated by a balance of activating and inhibitory receptors. ActRs and adhesion molecules help to form an immunologic synapse with target cells, with directional degranulation of lytic granules ^[30]^.

### 2.2 Metabolic changes with cytokine stimulation

Ex vivo cytokine stimulation of NK cells leads to different metabolic changes based on the stimulation length and type. Murine NK cells briefly (4 hours) stimulated with combinations of IL-12/IL-15 or IL-12/IL-18 produce robust IFN-γ protein. However, there are no detectable changes in the rate of OXPHOS or glycolysis, measured as oxygen consumption rate (OCR) and extracellular acidification rate (ECAR), respectively ^[17]^. The findings that inhibiting metabolism with OXPHOS inhibitor oligomycin, glucose-free media, or glycolytic inhibitor 2-deoxyglucose (2-DG) decreased intracellular ATP but not IFN-γ production demonstrated that IL-12/18 stimulation was independent of these metabolic pathways. Even cells cultured with 2-DG, while blocking other major mitochondrial OXPHOS fuels (glutamine or fatty acids), produced IFN-γ ^[17]^. These results suggest that in response to brief cytokine activation, NK cells have incredible metabolic flexibility allowing them to proceed with one of their main functions—IFN-γ production.

By contrast, longer-term (12–24 hours) culture of murine NK cells with IL-2/IL-12, which signals similarly to IL-12/IL-15 based on the shared receptor usage of the CD122 and CD132, increased mitochondrial metabolism and glycolysis, primarily via mammalian target of rapamycin complex 1 (mTORC1) ^[31]^. The Finlay laboratory elegantly elucidated factors important for these metabolic changes in murine NK cells expanded with IL-15 and then activated with cytokines (IL2/IL12). Glycolysis is necessary for NK cells effector function, as demonstrated by reduction of IFN-γ and granzyme B (Gzmb) when cultured with 2-DG or rapamycin during cytokine stimulation ^[31]^. However, glucose-derived pyruvate enters the mitochondria but does not proceed the classical TCA cycle. Instead, NK cells upregulate the citrate-malate shuttle (CMS), which exports glucose-driven mitochondrial citrate to the cytosol, where it is converted to malate and returned to mitochondria to fuel the electron transport chain (ETC), mediated by the transcription factor Srebp ^[32]^. In the cytoplasm, citrate is converted into acetyl-CoA and malate, with cytosolic acetyl-CoA used for lipid synthesis or protein acetylation. Inhibition of CMS machinery led to impaired NK cell OXPHOS and proliferation ^[32]^. This bypass of the TCA cycle with prolonged cytokine activation is supported by the finding that the absence of glutamine, which can fuel the TCA cycle, or inhibition of glutaminase, an enzyme required to utilize glutamine, did not alter OXPHOS ^[33]^. This coordinated shift in metabolism is partially regulated by amino acid import by SLC7A5 (L-amino acid transporter). Amino acids maintain cellular meylocytomatosis oncogene (cMyc) levels and regulate metabolism and thereby NK cell effector function, including IFN-γ and Gzmb production and target killing, in response to cytokine stimulation ^[33]^.

Together, these and other studies in murine NK cells demonstrate that the metabolic dependence of NK effector function varies by the type and duration of cytokine stimulation. Although short-term stimulation has minimal effects, longer-term activation enhances glycolysis and amino acid uptake, which can affect NK cell cytokine production and cytotoxic machinery. Understanding of the metabolic pathways involved in NK effector functions may also provide immunotherapy targets that are relevant for tumors but not NK cells. For example, combining a glutaminase inhibitor with NK therapy strategies might specifically impair the metabolism of tumor cells, while sparing NK cell function.

Human NK cells can be broadly subdivided into two populations. In general, CD56^bright^ NK cells produce more cytokines with activation, whereas CD56^dim^ NK cells have stronger cytotoxic activity, including antibody-dependent cytotoxicity ^[34]^. Studies have identified potential differences in metabolism in these subsets when stimulated with cytokines ^[35]^. Keating et al ^[35]^ demonstrated that fresh CD56^bright^ cells expressed higher levels of metabolic transporters (GLUT1, CD98, CD71) and increased mTOR-dependent glycolysis on IL-12/15 stimulation compared to CD56^dim^ cells. Overnight IL-12/15 stimulation in the presence of oligomycin reduced IFN-γ production in both subsets, but had no effect on Gzmb, suggesting that glycolysis and OXPHOS are essential for maximal IFN-γ production by CD56^bright^ cells. In contrast, Surace et al ^[36]^ found that fresh CD56^dim^ cells express more GLUT1. RNAseq and extracellular flux data suggested that cytokine-stimulated CD56^dim^ cells upregulated glycolysis and OXPHOS, but relied more on OXPHOS. Collectively, these studies demonstrate metabolic changes with prolonged cytokine stimulation of human NK cells. Further investigation is needed to define these changes, potentially due to the tremendous variability in human health and body composition. For example, obesity alters human NK cell activity, reducing the efficiency of immunotherapy, highlighting the importance of studying NK cells in individuals with different health conditions, and even personalizing therapeutic approaches to different metabolic states such as obesity and diabetes ^[37–39]^.

TGFβ is a well-known cytokine that inhibits NK cell function, including proliferation, cytotoxicity, and IFN-γ expression ^[40,41]^. TGFBR-deficient murine NK cells are more sensitive to IL-15 signaling and express higher T-bet, cytotoxic machinery, and metabolic receptors at baseline. Similar to the mouse, TGFβ inhibits glycolysis and mitochondrial metabolism in human NK cells ^[42]^. Therefore, blocking the TGFβ signaling pathways by gene editing or inhibitor is considered as an immunotherapy strategy to retain effector function of NK cells ^[43]^.

### 2.3 Metabolic requirements with receptor stimulation

NK cell recognition of tumors is dependent on ActR stimulation, thus an understanding of how metabolism alters these signals is important for immunotherapy. The structure of NKRs differs in mouse and human, but serve a similar function, an intriguing example of convergent evolution ^[44]^. Humans express killer cell immunoglobulin-like receptors (KIRs), while mice use the Ly49 family of C-type lectin-like receptors. Despite differences in structure and binding, these receptors recognize MHC and have similar signaling pathways and function as inhibitory and activating receptors ^[6]^. Both species also share NKG2 C-type lectin receptors, NKG2A/C/E, which partner with CD94 to recognize non-classical MHC, with NKG2A inhibiting NK cell function, and NKG2C and NKG2E partnering with DAP12 for an activating signal ^[45]^. NKG2D functions as an activating receptor in mouse and human and recognizes stress-induced ligands, Rae-1 in mouse, and MICA and MICB in human. These ligands are widely expressed by primary tumors but not by healthy cells, and NKG2D-mediated recognition is particularly relevant for cancer therapies ^[46]^. Activating natural cytotoxicity receptors (eg, NKp46, NKp44, NKp30) and Fc-binding receptors (CD16) stimulate NK cells in both species ^[47]^.

We demonstrated that short-term (6 hours) stimulation of murine NK cells through ActR Ly49D or NK1.1 did not alter ATP production, OXPHOS or glycolysis rate ^[17]^. Unlike cytokine stimulation, inhibition of OXPHOS or glycolysis during short-term receptor stimulation dramatically reduced IFN-γ production. In contrast, glycolysis but not OXPHOS affects cytotoxicity after Ly49H stimulation ^[48,49]^. Human NK cells upregulate both glycolysis and OXPHOS when activated with NKG2D and CD16 for a short term (4–6 hours). IFN-γ production by human NK cells also decreased with inhibition of glycolysis or OXPHOS, while cytotoxicity was only dependent on glycolysis ^[50]^, similar to our studies in the mouse ^[17]^. Human NK cells cultured in glucose-free media overnight had impaired degranulation, Gzmb production, and FasL expression ^[50]^. These findings suggest that strategies to overcome glucose requirements for NK ActR stimulation are required, and that adoptively transferred NK cells in the context of immunotherapy may not have efficient effector activity with ActR stimulation in glucose restricted areas such as the TME.

### 2.4 IL-15 primed NK cells: major metabolic shifts

IL-15 provides a critical signal for NK cell development, homeostasis, expansion, and activation in vivo ^[17,51]^. IL-15–based therapies are of interest to expand/activate NK cells ^[7]^. A common approach to studying NK cells in vitro is to first expand cells with IL-15 to obtain increased cell numbers for experiments, however this culture significantly changes NK cell physiology and metabolism. At high doses in vitro, IL-15 culture leads to NK cell “priming”, characterized by proliferation, arming of cytotoxic machinery, and enhanced effector function ^[8,29,52]^. IL-15 priming also induces mTOR and increases glycolysis and OXPHOS ^[8,17,52,53]^. However, NK cells primed in the presence of a glycolytic inhibitor failed to proliferate or upregulate cytotoxic machinery and ability to kill, suggesting that glycolysis is required during IL-15 stimulation ^[48,53]^. Intriguingly though, once NK cells are effectively primed, they acquired metabolic flexibility in vitro and in vivo. IL-15 primed NK cells exhibited a shift to transcriptional regulation of IFN-γ with ActR stimulation, no longer dependent on OXPHOS or glycolysis as in naive cells ^[17]^. In vivo IL-15 primed NK cells have increased glycolytic capacity. However, in vivo primed NK cells were resistant to subsequent glycolytic or mTORC1 inhibition with MCMV infection, where they exhibited efficient antiviral function and protected hosts, despite metabolic inhibition that causes impaired NK cell killing and fatal infection in naive hosts ^[48]^.

These data suggest that IL-15 priming of NK cells alters NK metabolism and leads to an enhanced effector state in which cells are more resilient to metabolic perturbations. This is intriguing for immunotherapy approaches, since NK cells primed with high-dose IL-15 may obtain sustained metabolic flexibility allowing them to retain their effector function in the TME. Although Felices et al ^[54]^ showed that continuous IL-15 exposure can cause human NK cell exhaustion in culture, further studies on the best approaches to using IL-15 priming for metabolic flexibility, such as timing of therapy, in patients are required.

### 2.5 Adaptive functions of NK cells

While categorized as innate lymphocytes, NK cells can establish immunologic memory, including specific recall response to antigen and nonspecific memory-like function after cytokine activation, akin to “trained immunity” in macrophages ^[55–62]^. Specific adaptive functions of NK cells have been demonstrated following infection or administration of haptens ^[61,63,64]^. In C57BL/6 mouse, murine cytomegalovirus (MCMV) infection induces cell surface expression of the virally-encoded glycoprotein, m157, which is recognized by NK cells via the germline-encoded activating Ly49H receptor ^[65]^. Activated Ly49H+ NK cells expand and kill virally-infected cells, followed by contraction and maintenance of a pool of Ly49H+ memory NK cells with enhanced effector function ^[66]^.

Metabolic regulation of MCMV-induced NK cell memory has been demonstrated in several studies. Ly49H+ cells had an increase in mitochondrial-associated reactive oxygen species (ROS) during initial NK expansion, which gradually decreased during contraction-to-memory ^[67]^. Removal of dysfunctional mitochondria by autophagosomes was important for this process, and treatment with rapamycin, which induces autophagy, during the contraction phase increased induction of NK memory.

Glycolysis and OXPHOS are both important for initial NK expansion and memory during MCMV infection. In a genetic model of inducible NK-specific *Cox10* deletion, a key component of complex IV of the mitochondrial ETC, loss of Cox10 did not impair NK cell differentiation, homeostasis, or IL-15 driven proliferation ^[49]^. However, receptor-mediated activation of NK cells was impaired, and Ly49H+ NK cells were unable to efficiently expand during MCMV infection. Sheppard et al ^[68]^ used a constitutive NK-specific model of *Ldha* deletion, inhibiting aerobic glycolysis, and observed that NK homeostasis was maintained, but there was impaired MCMV-induced proliferation, ActR signaling and cytotoxic function. Together, glycolysis and OXPHOS are essential for antigen-driven proliferation during viral infection, but there are other mechanisms that can compensate to maintain NK cell differentiation and homeostasis. These models highlight the ability of cells to adapt their metabolic machinery when needed, for example, the ability of cells with acute deletion of *Cox10* to survive and even differentiate/mature, but that the “effector” functions of NK cells require greater metabolic demand.

Humans infected with human cytomegalovirus (HCMV) develop a pool of adaptive NK cells characterized by high expression of NKG2C, CD57, and KIRs ^[69]^. These “adaptive” NK cells isolated from HCMV-seropositive donors have heightened glycolysis and OXPHOS accompanying higher mitochondrial membrane potential compared to conventional NK cells from HCMV-seronegative donors, suggesting that increased cellular metabolism supports adaptive NK cell expansion and survival ^[36,70]^. Intriguingly, these cells not just have better protection against viruses, but also cancer. Adaptive NK cells exhibited higher cytokine production in response to tumor cells and mediate relapse protection, and they are being targeted for immunotherapy ^[71–73]^.

NK cells activated overnight with IL-12, IL-18, and IL-15 exhibit an enhanced effector state characterized by rapid proliferation, enhanced IFN-γ production and killing in response to multiple stimuli including cytokines, receptors, and tumors that persists for weeks to months ^[10–12,55,74–76]^. These cytokine-induced memory-like (CIML) NK cells have shown promise as immunotherapy in early clinical trials ^[4,9,10]^. Human CIML NK cells have higher expression of CD98 and GLUT1 with enhanced glycolysis, and activated NK cell showed better target cell killing in the presence of 2-DG compared to naive NK cells, meaning CIML NK cells might be able to maintain better antitumor activity in TME ^[77]^.

Together, these studies demonstrate that features of NK cell memory are regulated by metabolism. Additional work to understand mechanistically how metabolism regulates the formation of the memory program may provide insight into harnessing these cells for targeted immunotherapy. For example, a better understanding of how nutrients including glucose and immunosuppressive cytokines affect adaptive NK cells function. In addition, it is unknown what metabolic changes NK cells undergo after adoptive immunotherapy in patients.

## 3. Metabolic changes in the tumor microenvironment and implications for immunotherapy

**Figure 1.**
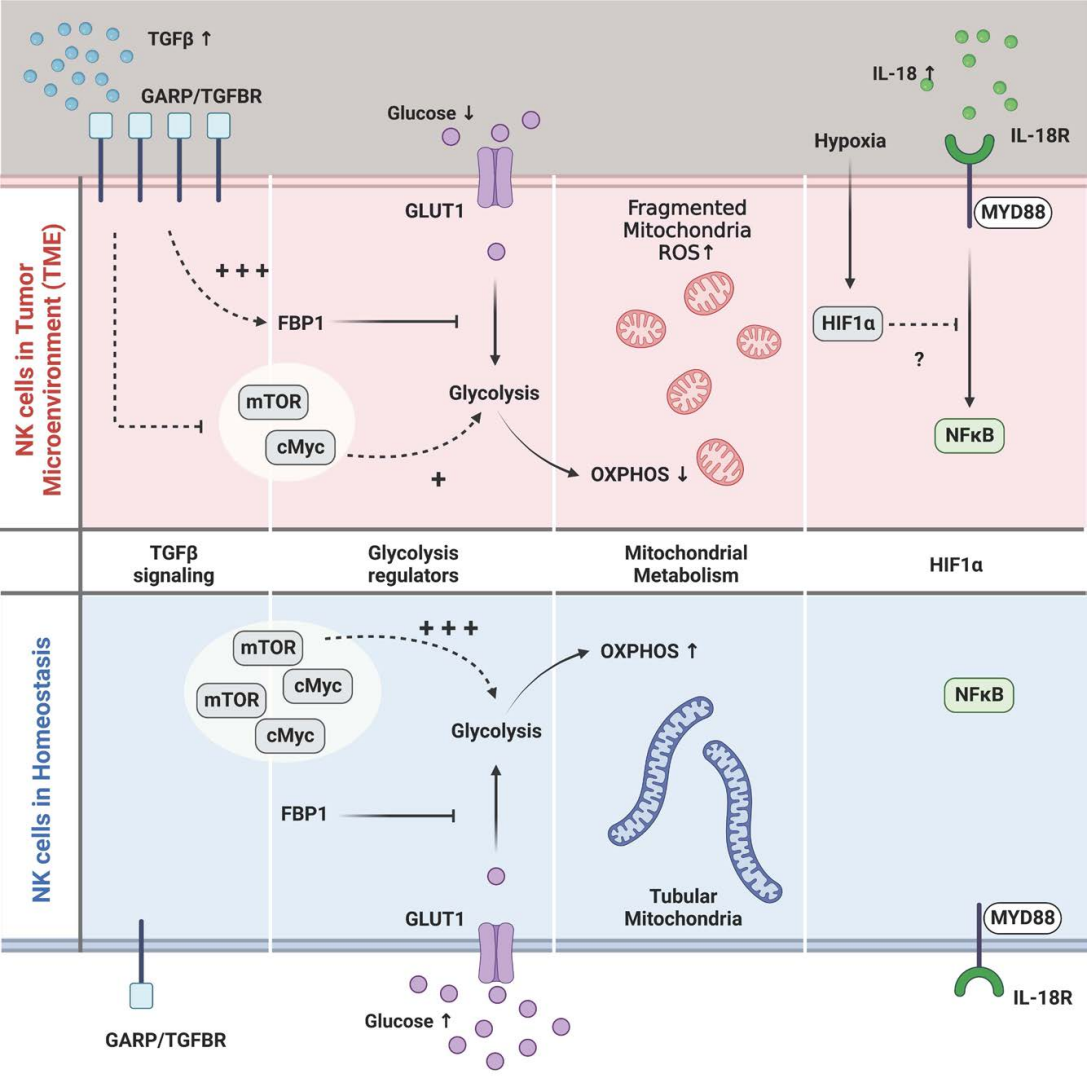
Overview of potential perturbations of NK cell metabolism in the TME. The TME creates a distinct milieu characterized by low glucose, hypoxia, and high levels of the anti-inflammatory cytokines including TGFβ. NK cells in the TME express more of the latent TGFβ receptor, GARP. Binding of GAPR to its ligand suppresses glycolysis through upregulation of FBP1 and inhibition of glycolysis regulators including mTOR and cMyc. NK cells rely upon glucose to fuel OXPHOS, including the citrate-malate shuttle, and low glucose concentration in TME likely impairs NK cell OXPHOS. Tumor-infiltrating NK cells have been shown to have fragmented mitochondria distinct from the tubular shape in the periphery. Fragmented mitochondria can lead to elevated ROS and apoptosis. The transcription factor HIF1α, induced in hypoxic conditions, was shown to be a negative regulator of NK cell antitumor function, in part due to suppression of IL-18 signaling. cMyc: cellular myelocytomatosis oncogene, FBP1: fructose-bisphosphatase 1, GARP: latent TGFβ receptor glycoprotein-A repetitions predominate, HIF1α :hypoxia-inducible factor 1-alpha, mTOR: mammlian target of rapamycin, NK: natural killer, OXPHOS: oxidative phosphorylation, ROS: reactive oxygen species, TME: tumor microenvironment. Figure was created with BioRender.com.

Because the TME influences metabolic reprogramming and functioning of immune cells, understanding how TME changes immune cell metabolism is critical for optimal therapies. TME disrupts the antitumor function of immune cells in a variety of ways, including metabolic modification through high utilization of available energy sources and production of metabolites and cytokines that alter NK metabolism (Figure 1), and NK cells from mice and humans have been shown to exhibit changes in metabolism in the TME.

Hypoxia in the TME due to increased oxygen usage by tumors has the potential to impact NK cell function as OXPHOS is a main pathway used to fuel NK function ^[78]^. The transcription factor hypoxia-inducible factor 1-alpha (HIF1α) regulates expression of genes important for glucose metabolism, cell proliferation, survival, and angiogenesis, and is induced in hypoxic environments as a compensatory mechanism ^[79]^. Interestingly, HIF1α-deficient NK cells led to improved tumor clearance and increased expression of activation markers and effector molecules ^[80]^. In this model, an IL-18-NFκB signature compensated for HIF1α loss, suggesting that HIF1α restrains IL-18 signaling, which potentially explains the enhanced NK cell effector response.

Human NK cells appear to be more vulnerable to oxidative stress than T and B cells, with CD56^dim^ cells more susceptible than CD56^bright^ cells ^[81]^. Tumor-infiltrating NK cells from breast and liver tumor patients had smaller, fragmented mitochondria, associated with ROS and mitophagy, while peripheral NK cells had tubular mitochondria ^[82,83]^. Overexpression of PRDX1, an antioxidant enzyme, in CAR-modified NK-92 cells increased killing of tumor cells and survival under oxidative stress ^[81]^. This suggests that one possible way to mitigate oxidative stress might be IL-15 priming, which upregulates expression of PRDX1.

Slattery et al ^[82]^ demonstrated that breast cancer patients had fewer tumor-infiltrating CD56^bright^ NK cells with decreased IFN-γ production and poor cytotoxicity. This impaired function correlated with reduced metabolic receptors expression (CD71, CD98), glycolytic capacity and OXPHOS. Tumor-infiltrating NK cells from patients expressed the latent TGFβ receptor glycoprotein-A repetitions predominate (GARP), and treatment with anti-GARP in vitro increased OXPHOS and restored effector function, suggesting that TGFβ in the TME was altering NK cell function. TGFβ inhibits the metabolism of mouse NK cells by increasing the expression of fructose-bisphosphatase 1 (FBP1), which is a negative regulator of glycolysis ^[84]^. Consistent with the importance of glycolysis for NK cell tumor control, deleting *LDHA* in NK cells also impaired tumor clearance through impaired IFN-γ production and cytotoxic capacity ^[68]^.

Another example of a tumor-derived product that can alter NK metabolism is lactate. In mouse models, elevated extraceullar levels of lactate produced by tumor cells impairs NK cell cytokine production, cytotoxic activity, and energy metabolism ^[78,85,86]^. NK cells from the mice challenged with melanoma or pancreatic cancer cells that were LDHA deficient, and therefore did not secrete high levels of lactate, had enhanced IFN-γ and Gzmb production and tumor killing ^[85,86]^.

These studies support the need for development of strategies to allow NK cells to persist and function in the TME. Apart from IL-15 priming, there are several additional potential strategies to regulate NK metabolism for successful immunotherapies ^[15,87–90]^. For example, CAR-NK cells expanded better and were less exhausted when incubated with IL-21-expressing feeder cells by upregulating metabolic receptors and glycolysis ^[91]^. Similarly, CAR-NK cells generated from CIML NK cells exhibit enhanced functional responses, which may be due to the advantage of upregulated metabolic receptors and glycolysis-skewed metabolism of CIML NK cells ^[77,92]^. CAR T cells trained in glucose-limited conditions improved persistence and response ^[93]^. However, low glucose availability reduces the viability and response of NK cells ^[17,31,37,48,50]^, and it remains to be seen whether CAR-NK cells exposed to glucose restriction can adapt to TME.

Combining NK immunotherapy with drugs that promote or restore NK metabolic function is an approach that may help in the setting of immunotherapy. For example, ex vivo expanded human and murine NK cells treated with glycogen synthase kinase 3 (GSK3) inhibitors, which prevent cMyc protein from being degraded ^[94]^, had enhanced cytokine production and antitumor activity against ovarian cancer and leukemia in vitro ^[95,96]^. Another example of metabolic manipulation was shown in a murine model of lung cancer, where NK cells from lung of tumor-bearing mice were stimulated ex vivo with IL-2/12 in the presence of an FBP1 inhibitor. This stimulation led to an increased NK cell glycolytic rate, and improved NK cell tumor clearance when cells were adoptively transferred to lung cancer-bearing mice compared to cells stimulated in the absence of inhibitor ^[84]^.

In summary, including nutrient depleted environments, the presence of anti-inflammatory cytokines such as TGFβ and hypoxia are all examples of ways in which the TME can affect NK cell metabolism (Figure 1). Design of NK cell therapies should consider strategies to restore NK cell function in these challenging environments.

## 4. Conclusions

Immunometabolism is crucial for immune cell function and can impact immunotherapy. A better understanding of how metabolism and the availability of metabolic fuels affect NK cell function is important to accelerate development and refine NK cell-targeting treatment approaches. Studies in mice and humans have revealed important metabolic pathways that sustain NK cell effector functions in the context of tumors and viral infection, as well as potential strategies for “arming” NK cells for these environments such as IL-15 priming. A further understanding of the metabolic demands of NK cell immunotherapy has the potential to allow for enhanced therapies for treatment of cancer and viral infection.

## Author contributions

HS and MAC wrote and edited the manuscript.

## Conflicts of interest

The authors declare no conflict of interest.

## Funding

This work was funded by NIH R01AI127752, “Metabolic Regulation of Natural Killer Cell activation”.

## Acknowledgments

The authors thank InPrint: A Scientific Communication Network at Washington University in St. Louis for editing assistance. Figure was created with Biorender.com.
